# Mr. Vandier's Observations on the Kilburn Waters

**Published:** 1801-09

**Authors:** F. N. Vandier

**Affiliations:** Great Mary Le Bone Street


### *To the Editors of the Medical and Phyſical Journal.*

Gentlemen,

I Was in hopes that ſome of your learned correſpondents would take the trouble of offering to the younger part of your readers, ſome Remarks on "An Analyſis of the Kilburn Mineral Waters, by John Bliſs, Member of the Royal College of Surgeons in London," publiſhed in the Medical Review for July, 1801: Having been diſappointed in my expectations, I am induced to trouble you with this letter, leſt ſuch an analyſis paſs entirely unnoticed.

Every perſon acquainted with analytical chemiſtry, will readily admit the difficulty of determining, in the preſent ſtate of our knowledge, with any degree of certainty, the abſolute quantity of the ingredients which enter into the compoſition of any ſubſtance. This is true of concrete metallic and earthy ſubſtances fubftances, but deliquefcent falts, and mineral waters, are analyfed with ftill greater difficulty. All authors agree on the impoffibility of afcertaining the precife quantity of the various matters fufpended or diffolved in waters, on account of the fmall portion contained in a given weight of the liquid, and on account of the nature of thefe matters. But, if we cannot arrive at fuch fatisfactory refults as we could wifh, we may generally difcover pretty accurately, what fubftances do, and what do not, enter into their compofition.

It was certainly laudable in Mr. Blifs, to undertake the experiments he has publifhed; and for my own part, I am very thankful to fuch gentlemen as employ their fkill and abilities in promoting the ftudy and knowledge of chemiftry, perfuaded as I am, that it is a branch of fcience too much neglected by, though indifpenfably neceffary, to medical men.*

But has not that gentleman been rather unfortunate in his analyfis? I do not mean to enter into a long criticifm, nor to follow the author in all his fteps. I cannot help however remarking, that the account appears to me rather confufed, and far diftant from the concifenefs and perfpicuity of the analyfes publifhed by Meffrs. Vauquelin, Klaproth, Prouft, &c.

It appears to me alfo, that Mr. Blifs employs very extraordinary expreffions; for inftance, what does he mean by "Strontian lime water?" Did he ufe a folution of the two earths in water? or does he mean to fay, that he ufed a folution of Strontian in water, as we ufe fometimes a folution of lime in water? In this cafe, Strontian water would have been fufficient and more correct; the appellation "Strontian lime," is, to fay no more, a barbarifm.

According to Mr. Blifs, there exifts in the Kilburn water, befides other matters:

Carbonate of lime, and of magnefia.

Muriat of lime, of magnefia, and of foda.

Sulphat of lime, of magnefia, and of foda.

I had been taught that the muriat of lime was decompofed by the fulphat of foda and of magnefia; and having the fingular advantage of affifting a learned philofopher† in his chemical experiments, I had often witneffed fuch a decompofition; confequently, I was greatly aftonifhed at the refult of Mr. Blifs's analyfis ftated as above. After his pofitive affertion, I could not, at firft, but diftruft my memory, and have recourfe to experiments; I therefore poured fome fulphat of foda into a folution

* I may, perhaps, enlarge on this fubject at fome future opportunity.

† Richard Chenevix, Efq. F. R. S. and M. R. I. A.

of muriat of lime; the effect was, as indeed might have been expected, a copious precipitate of fulphat of lime, the muriat of foda remaining in folution.

I then poured fome fulphat of magnefia into a folution of muriat of lime, and there again a copious precipitate of fulphat of lime was produced. Both folutions being faturated the refult was more ftriking, and prefented what was formerly called *miraculum chemicum*, a white folid body refulting from the mixture of the two liquids. I thought thefe two fimple experiments quite fufficient to prove the conclufions of Mr. Blifs to be erroneous. I, however, confulted my celebrated countryman, Prof. Fourcroy, and I read: "All the fulphats, except the calcareous, are decompofed by the muriat of lime, by double elective attraction. Sulphat of lime is formed and conftantly precipitated from the folutions of thefe falts mixed together, and there remain in the fupernatant liquor foluble muriats, the bafes of which differ, according to the fpecies of fulphats which have been decompofed." Syftème des Connoiffances Chimiques, Tome iii. p. 194. Again, "Sulphat of magnefia is decompofed by carbonat of lime; carbonat of magnefia and fulphat of lime are formed. Sulphat of magnefia is decompofed by muriat of lime; muriat of magnefia and fulphate of lime are formed. Sulphat of foda is decompofed by muriat of lime; muriat of foda and fulphat of lime being formed. Id. Tome iv.

Treating of mineral waters, the learned Profeffor obferves, "It would be almoft fuperfluous to fay, that fome falts exclude one another, as the fulphat of foda and of magnefia with the nitrat and muriat of lime, the calcareous falts with the carbonate of foda, &c." Id. Tome ix. p. 298.

I muft conclude by faying, that, if Mr. Blifs cannot reconcile his analyfis with thefe facts and quotations, we are warranted to confider it as erroneous, fince it is afferted that he found in the waters, falts which cannot poffibly exift together.

*Great Mary Le Bone Street,*
*August* 14, 1801.

I am, &c.

F. N. VANDIER.

*Remarks*

